# Potential of Using Amazon Natural Fibers to Reinforce Cementitious Composites: A Review

**DOI:** 10.3390/polym14030647

**Published:** 2022-02-08

**Authors:** Thuany E. S. de Lima, Afonso R. G. de Azevedo, Markssuel T. Marvila, Verônica S. Candido, Roman Fediuk, Sergio N. Monteiro

**Affiliations:** 1 LAMAV—Advanced Materials Laboratory, UENF—State University of the Northern Rio de Janeiro, Av. Alberto Lamego, 2000, Campos dos Goytacazes 28013-602, RJ, Brazil; thuanylima.es@gmail.com; 2 LECIV—Civil Engineering Laboratory, UENF—State University of the Northern Rio de Janeiro, Av. Alberto Lamego, 2000, Campos dos Goytacazes 28013-602, RJ, Brazil; 3 CRP—Rio Parnaíba Campus, UFV—Federal University of Viçosa, Rodovia BR-230—Km 7, Rio Parnaíba 38810-000, MG, Brazil; markssuel@hotmail.com; 4 Conselho Superior de Ensino e Pesquisa, UFPA—Federal University of Pará, Rodovia BR-316—Km 7500-9000, Centro, Ananindeua 67030-007, PA, Brazil; scarpini@ufpa.br; 5 Polytechnic Institute, Far Eastern Federal University, 690922 Vladivostok, Russia; roman44@yandex.ru; 6 Department of Materials Science, IME—Military Institute of Engineering, Square General Tibúrcio, 80, Rio de Janeiro 22290-270, RJ, Brazil; snevesmonteiro@gmail.com

**Keywords:** natural fibers, Amazonian fibers, cement-based composites

## Abstract

The engineering application of natural lignocellulosic fibers (NLFs) has been intensifying all over the world due to their low cost and abundance, as well as their being eco-friendly and presenting favorable technological properties in polymeric and cementitious composites. Brazil, especially the Amazon region, owing to its climate and geographic position, has an abundant variety of NLFs that are still unexplored with great potential for use in various composite materials and applications such as civil construction, automobile parts and armor. Therefore, this review aims to establish a parallel between the technological properties of cementitious composites reinforced with Amazon NLFs, both in fresh and hardened states, and to analyze, compare results and contribute to a better understanding of the similarities and differences between the types of reinforcements. A relevant contribution of this review is the possibility of improving knowledge about Amazon NLFs, showing their potential for application in eco-friendly materials, in addition to contributing to studies with new NLFs not yet applied in composite. For this, it was necessary to carry out a literature survey on the physical, chemical and mechanical properties of cementitious composites reinforced with NLFs, in addition to analyzing case studies involving fibers such as curaua, açai, bamboo, jute and sisal. It can be concluded that the physical and chemical characteristics of the Amazon NLFs directly influence the technological properties of cementitious compounds, such as mechanical strength and water absorption. However, there might be a need for surface treatment aimed at improving adhesion and durability of the cementitious composite. Finally, some suggestions for future research work are highlighted in order to show the need to continue investigations on the application of Amazon NLFs in cementitious composites.

## 1. Introduction

Composite materials are developed with the aim of forming a new, higher quality product with special or better properties than the individual materials alone. In this way, a large number of new materials can be developed from the combination of other materials [1].

These types of materials consist of two phases: matrix and reinforcement. In general, matrix is the continuous phase, while reinforcement is the dispersed phase. The matrix protects the reinforcement, preventing the dispersed material from coming into contact or reacting with the external environment, thus preventing the reinforcement from being affected by mechanical abrasion or chemical reactions from external agents. The reinforcement, in turn, is responsible for distributing the load applied to the material, allowing the matrix to withstand the efforts [2].

Composites are today used for very diverse applications including biomedicine [3], the civil construction sector [4] and aerospace [5]. The matrix can be produced using metallic [6,7], ceramic [8,9] or polymeric materials [10,11], while the reinforcement can be formed with particles or fibers, being responsible for the main mechanical properties, such as strength and ductility of the composite.

In the specific case of cementitious composites, as the name suggests, the matrix is composed of cement-based materials, which can be obtained through the application of geopolymer pastes, mortars or concrete [12,13]. The concrete or the mortar are themselves composite materials; the behavior of cementitious composites is complex and can have significant variations depending on the type of filler used as reinforcement. It is known that cement-based materials have excellent compression properties and fragile behavior [14,15]. For this reason, the use of with a filler has the objective of improving the tensile strength and ductility [16,17].

The aims of this review are to compile and discuss important research that has used Amazon natural lignocellulosis fiber (NLFs) as reinforcement for cementitious materials, approaching their advantages and disadvantages in terms of applications and challenges in the processing and exploitation of these natural resources, which have high potential for use in the development of eco-friendly materials. As specific objectives of this research, we can quote: (i) improvement of the discussions about the feasibility or not of the application of Amazon NLFs in cementitious materials; (ii) understanding of the particularities related to natural fibers from the Amazon region; (iii) deepening of the main mechanisms of NLF interaction (reinforcement) with the cement matrix; and (iv) discussion about the importance of the treatment of natural fibers for durability and applicability in cementitious materials. In addition, the in-depth study of these research works will contribute to the advancement of the state-of-the-art knowledge on the subject, and will strengthen the potential for sustainable exploitation of Amazon NLFs. Another point highlighted is that the Amazon region has enormous biodiversity and great potential for the emergence of NLFs that have not yet been applied in engineering composites. Therefore, this work can contribute to the study of new fibers, enabling the comparison of their properties with those of fibers currently studied, enabling their exploitation. Major problems in the application of NLFs in cementitious materials, concerning to their durability and variability, are also explored in this research.

### Advantages and Disadvantages

Fibers can be classified as natural, such as vegetable, mineral (asbestos) [18,19] and siliceous (such as wollastonite) [20,21], or synthetic fibers, such as steel [22,23], glass fibers [24,25], carbon [26,27] and polymeric fibers (nylon, polyester, PVA) [28,29]. The main advantages of applying natural plant fibers are the availability of the material, as they are abundant in nature [30]. In addition, agro-industrial residues from NLFs can be used, such as those from coconut, banana or sisal farms [31]. This type of natural fiber is environmentally friendly, as the production of the material does not emit polluting gases or consume mineral resources, in addition to the material being biodegradable [32]. Another benefit is the low cost of NLFs compared to synthetic fibers. In fact, due to their easy cultivation or natural extraction, they do not require very sophisticated technologies or high production costs [33,34]. For exemple, sisal fibers, the most expensive NLFs studied by Shahinur and Hasan [35], cost 0.45 USD/kg, representing a 43.75% lower price in relation to steel fibers. The density of the composite is another advantage that deserves to be highlighted, as natural fibers are lighter and less dense than synthetic fibers [36,37].

One main disadvantage of these composites is the fact that the properties of NLFs are more heterogeneous than synthetic fibers. As they come from a controlled industrial process, synthetic fibers have well-defined properties and characteristics [38,39]. NLFs, on the other hand, are subject to the weather and climatic variation in nature, changing their properties depending on the geographic location, time or season of the year, and other environmental conditions. Another problem associated with NLFs applied in cementitious composites is related to the durability of these materials [40,41]. The NLFs are chemically composed of organic materials, such as cellulose, lignin and hemicellulose, which are also the compounds responsible for the formation of microfibrils that reinforce the material matrix [42]. However, in an alkaline environment, as is the case with the cementitious matrix, these fibers undergo degradation due to chemical decomposition of lignin and hemicellulose, compromising the durability of the material [43,44].

Thus, based on the available literature on the subject, this review aims to establish a parallel between the technological properties of cementitious composites reinforced with Amazon NLF in both fresh and hardened states, seeking to analyze and compare their results as well as contribute to a better understanding of the similarities and differences between the types of reinforcements.

## 2. Anatomy of Amazon Natural Lignocellulosic Fibers (NLFs)

To understand the behavior of NLFs, it is necessary to have knowledge of their morphology and chemical composition as well as their physical and mechanical properties.

There are a wide variety of plant fiber morphologies, which depend on the environmental conditions in which these fibers develop, such as the soil, water, air and sunlight [45]. As environmental conditions are not the same worldwide and in all seasons of the year, these variations influence the growth of plants and consequently their chemical, physical and mechanical properties [46]. However, despite the fact that NLFs have various origins and are affected by the characteristics of the growing region, their morphological structures and the basis of their chemical compositions are very similar to each other [47]. 

**Figure 1:**
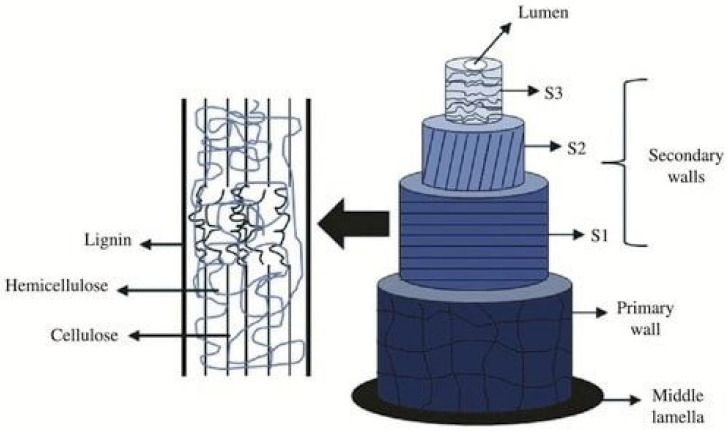
Structure of plant fiber [48].

The high tensile strength that NLFs might have is due to their hierarchical nature, where each fiber is individually formed by several fibrils, and each fibril is divided into three main parts: (i) the primary wall; (ii) the secondary wall (thicker and stronger—a product of three layers); and (iii) the lumen. The fibrils are linked together through the middle lamella, formed by hemicellulose and lignin [47]. Figure 1 illustrates the morphological structure of a plant fiber, in which the complexity of the fibril layers is highlighted.

The primary wall is the first layer deposited during cell growth. Its microfibrils are arranged randomly, not providing much resistance to this layer. The orientation of the microfibrils is called the microfibrillar angle, that is, the average angle at which the microfibrils are inclined in relation to the axis of the filament. Strength and stiffness are inversely proportional to the microfibrillar angle [49]. The first layer is thin, and surrounds the secondary wall, which, in turn, is composed of three layers (S1, S2 and S3). S2 is composed of microfibrils arranged in a helical shape, providing a lot of resistance to the layer, making S2 the thickest of the layers and generally responsible for the mechanical properties of the fiber. The transversely arranged central cavity is called the lumen, responsible for the fibers being hollow [50].

Each wall consists of cellulose, lignin and hemicellulose in the vast majority of layers, together with other components in smaller amounts, such as pectin, oils and waxes [51]. Table 1 shows the percentage of these components for different NLFs cultivated in the Amazon region, which are the subject of this review. To our knowledge the are the only Amazon NLFs currently applied as reinforcements of cementitious composites. Among these fibers, açai [52], curaua [53,54], guaruman [55] and piassava [56] are native to the Amazon region, while the others are only locally cultivated; bamboo [57,58], jute [53,59] and sisal [58,59] that are originally from distinct regions of the world, such as East Asia, Middle Asia, Africa and North America.

The chemical composition of NLFs has a great influence on their mechanical properties and durability. This is because cellulose, hemicellulose and lignin are the main compounds responsible for the strength, adhesion and degradation of fibers [2,46].

Cellulose is structurally the most important organic component, as it is more rigid and resistant, providing stability and strength to NLFs. It is a natural polymer and its molecules include glucose units linked together, forming long chains that, in a row, form microfibrils. However, cellulose is a semi-crystalline polysaccharide with a large amount of hydroxyls, making it hydrophilic. This characteristic increases the fiber’s water absorption, and with it, it loses strength and adhesion when wet [60]. 

Hemicelluloses are low molecular weight polysaccharides linked in short, branched chains. They help to incorporate the cellulose, functioning as part of the matrix, being linked to the microfibrils through hydrogen bonds. Hemicellulose has an open and completely amorphous structure, with hydroxyl and acetyl groups, making it water-soluble and hygroscopic. It is responsible for the biodegradation, microabsorption and thermal degradation of NLFs [61].

Lignin is an aromatic polymer with high complexity and irregular constitution; that is, it is amorphous. It gives the plant rigidity, working as a chemical adhesive within and between the NLF [46]. It works by helping to transport water in the fiber, as it is the compound that absorbs less water.

Finally, Table 1 shows the natural moisture content of the NLFs ranging from 10 to 18%. Moisture in the fibers occurs because they are plant cells in association. Therefore, drying the fibers in ovens should not be carried out at temperatures above 100 °C, at the risk of degrading the fibers due to the natural moisture present [53,62].

The physical properties of NLFs are determined by parameters such as the shape and dimensions of the microstructure of the filaments, the microfibrillar angle and the thickness of the walls of the internal structure. The smaller the microfibrillar angle, that is, the more aligned to the fiber, the greater its strength and rigidity; larger angles provide greater ductility [51]. 

With the use of X-ray techniques and the inclination of the microfibrillar angle, it is possible to verify whether the NLFs are in the form of a helix or spiral. Fiber shape is important because helix-shaped fibers tend to cluster at lower percentages than spiral-shaped fibers [63]. Therefore, the critical volume of these fibers changes depending on their shape and microfibrillar angle. In general, the greater the microfibrillar angle, the less the fiber tends to agglomerate and the greater the fiber content that can be used in cementing matrices without any loss of mechanical properties.

Fiber water absorption is a physical property with extremely high results, as shown in Table 2. This makes the behavior of cementitious composites with NLFs extremely problematic, due to two principles: (i) since the fiber absorbs too much water, the matrix cement may lack water for the cement hydration reactions to occur [52]; and (ii) for the high water absorption, it may internally form a water accumulation point that will make the compound weaker, and hence problematic [64,65]. Several durability problems presented by cementitious materials with NLFs occur due to this problem. On the other hand, if the water absorption of the fibers was too low, another problem would occur because the adhesion points of the efforts between the matrix and the reinforcement would not be formed [66,67]. These points occur due to the suction that the fibers cause in the matrix paste, generating bridges mainly caused by the formation of ettringite crystals, which maintain a strong bond between the fiber and the matrix [68,69].

Generally speaking, NLFs can be fibrillar or tubular [70,71]. Tubular fibers are molded inside and have a high adhesion to the matrix, superior to fibrillar fibers, which are massive, presenting less adhesion to the matrix and, consequently, lower mechanical resistance [71]. This pattern, however, must be carefully analyzed due to the absorption of water, which is greater in the tubular fibers, which can cause problems of durability. Furthermore, regions of internal water concentration or excessive suction of water from the matrix can occur, compromising the cement hydration and the final strength of the composite [70,72].

**Table 2:** Physical properties of Amazon NLFs.

Fiber Type	Density (g/cm^3^)	Diameter (μm)	Microfibrillar Angle (°)	Water Absorption (%)	Crystallinity (%)	Reference
Açai	1.4–1.7	110–120	-	-	-	[52]
Bamboo	0.60–1.1	10–40	2–10	145	57–62	[57,73,74]
Curaua	1.4	26–61	15	360	44–66	[53,59,75,76,77]
Guaruman	0.5–1.1	40–60	7.3–8.2	-	60–67	[55,78]
Jute	1.3–1.5	60–110	8	62	71	[79,80,81,82]
Piassava	1.4–1.6	200	16–35	34–108	25–29	[56,59,83,84,85,86]
Sisal	1.3–1.5	100–300	20	110–239	57	[53,63,79,83,87]

As shown in Table 2, NLFs have a low density. They also have an internal cavity, called the lumen, with a variable shape, and therefore display considerable variations in diameter along their length. This decreases the fiber density, acting as an enhancer of the acoustic and thermal insulation capacity, but also affects the tensile strength of the fibers [49,88].

Voids are also part of the structure of NLFs. The greater the degree of porosity, the greater the absorption of moisture by the fiber. The moisture content of the fibers influences the tensile strength, the degree of crystallinity and swelling, which facilitates fiber degradation in alkaline environments [89]. The other physical properties such as density and electrical resistivity are related to the internal structure and chemical composition of the fiber [51].

This last aspect is of great relevance since the NLF diameter also influences the mechanical properties. The diameter dimension depends on the lumen and varies according to the location of the studied fiber. On average, the tensile strength decreases with increasing fiber diameter [90]. As the tensile strength and modulus of elasticity tests depend on the cross-sectional area of the individual fiber, the most appropriate procedure would be to determine an average cross-sectional area to calculate more accurate stress values [76]. Table 3 shows the great variability of results found among the surveys for Amazon NLFs due to the variable cross-section along the fiber length.

Some authors [77,94] point out that there is a direct relationship between the strength of the composite and the diameter of the fiber used. Tomczak et al. [77] carried out this analysis using 20 mm-long curaua fibers with diameters from 26 to 61 μm. The authors observed that the increase in fiber diameter, in general, does not affect the breakage strain of the composite, which is around 4.5%, but reduces its breaking stress; that is, it reduces its strength at failure. In addition, the use of fibers with a smaller diameter favors an increase in the elasticity modulus of the material. Monteiro et al. [94] found an inverse hyperbolic correlation between the tensile strength and the diameter for curaua, sisal, ramie, jute, bamboo, coir, piassava and buriti fibers.

NLF orientation is another factor that causes direct impacts on the mechanical properties of composites, especially on long fibers [95]. Vegetable fibers, however, are generally classified as short fibers [96]. Furthermore, in cementitious matrices, the fiber orientation is almost always random [97], as the material production process, usually molding, does not allow the use of fibers in a preferred direction. Therefore, there is no direct relationship between fiber orientation and mechanical properties for cementitious matrices.

While the tensile strengths of Amazon NLFs are very high, as shown in Table 3, cement matrices have extremely low tensile strength, with values around 1.1–1.34 MPa in the case of mortars, for example [52,98,99]. This information highlights the need to use NLFs in mortars, as this is an ecologically correct and economically viable way to increase the strength of mortars. 

Regarding the mechanical property related to the modulus of elasticity when the matrix breaks, NLFs with a lower modulus of elasticity will not be mobilized instantly; there is an interval for them to start acting as reinforcement. On the other hand, fibers with a higher modulus of elasticity are mobilized immediately. However, as they are already subjected to a higher tension level, they may be closer to their ultimate tensile strength [100].

NLFs have great variability of properties, as observed in Table 1, Table 2 and Table 3, due to the different conditions to which they are subjected. These include plant cultivation, species, geographic origin, location of fiber in the plant and local climate as well as maturation, which involves growth processes that influence the fiber wall thickness, roughness, fiber adhesion to the matrix, lumen size and shape, porosity and microfibrillar angle; the extraction process, which can be by maceration or separation; the transport and storage of these fibers; and finally, the test conditions such as the speed rate, fiber moisture during the test, and cross section at different points evaluated [63]. In the specific case of fibers extracted from the Amazon region, this variation is more suggestive than the other fibers, since this region presents high climatic variation and the influences of several natural phenomena that alter local conditions. Currently, global phenomena such as the greenhouse effect have been spreading heat waves, cold and high rates of rainfall, which end up affecting local biodiversity [40], as can be seen in the Table 1, Table 2 and Table 3. This variation raises the discussion for the need for continuous studies and knowledge about these materials, justifying this review.

## 3. Interface between Fiber-Matrix

**Figure 2:**
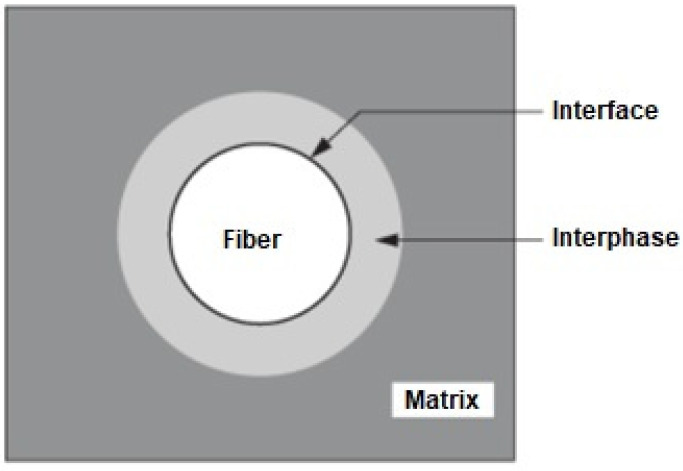
Interface and interphase concept [103].

The characteristics of strength, deformation and the types of ruptures to which the composite materials will be subjected depend mainly on the adhesion between the reinforcement and the matrix. Good adhesion between the fiber and the matrix reduces the size of the cracks, caused by the application of tension in the material [101]. The compatibility between fiber and matrix is closely linked to the concepts of interface and interphase of the material. The composite interface is a surface, practically without thickness, that joins the reinforcement and the matrix by adhesion mechanisms [102]. On the other hand, the interphase is the region that results from the physical-chemical interaction between the reinforcement and the matrix [103]. These concepts are illustrated in Figure 2. 

The importance of a good interfacial region between the fiber and the matrix is visualized when it is reflected in the increased integrity of the composites and the efficient transfer of the load to the fibers. The physicochemical characteristics of the interface are responsible for the mechanical properties of the composite as a whole. However, both interface and interphase help with the mechanical properties of the composite material, the main one being the adhesion of the material [104]. 

The great challenge in the processing stage of composite materials is to obtain adequate interfaces and interfaces between matrix and reinforcement. A weak interface bond can result in pullout of reinforcements and low mechanical strength. In contrast, a strong interface bond provides composites with greater strength but reduced toughness. To obtain both strength and high tenacity, the processing adjustments of the interface bond must be performed well [105].

To achieve better interfacial adhesion, the combination of materials must have the same properties. The adhesion between the matrix and the reinforcement can be attributed to: mechanical adhesion, electrostatic attraction, chemical bonds and interdiffusion of materials. Such characteristics will create a strong bond between the constituent materials of the composite [106].

Mechanical adhesion is mainly influenced by the roughness of the reinforcement material. In cases of composites that use fibers as reinforcement, the surface roughness of these plays an important role in increasing the bond between fiber and matrix [105]. When electrostatic attraction occurs between the matrix and the reinforcement, electrical charges are transferred from one to the other, leading to the formation of a negative charge on one substrate and a positive charge on the other. This generates an electrostatic attraction between the composite components [104]. Chemical adhesion is given by bonds of the electronic structure during the formation of the interface. The strength of this type of interaction depends on the type and number of chemical bonds per unit area [107]. Interdiffusion occurs via transfer of the atoms from the substrate to the molecular unit of the fibers, and vice versa. Sometimes this type of adhesion can be thought of as a tangle of molecules. In this case, the factors that control the resistance at the interface are the distances between each entangled molecule [108].

The binding of molecules through interdiffusion is essential for the strength of the composite, as strong bonds between molecules result in a better transfer of tensions from the matrix to the fibers. Thus, it can be seen that the bonds of molecules are closely related to the mechanical properties of the material, such as mechanical performance and type of fracture rupture [108,109].

Composite materials are considered anisotropic because their properties are verified in different ways depending on the position in which the stress is subjected on the material. An example that explains this characteristic and is linked to the reinforcement–matrix interface is the shear strength and tension. When load is applied to the material in the direction parallel to the interface, the material strength will be high. However, when the interface is subjected to tensional forces perpendicularly, the shear strength of the material will be low [110].

**Figure 3:**
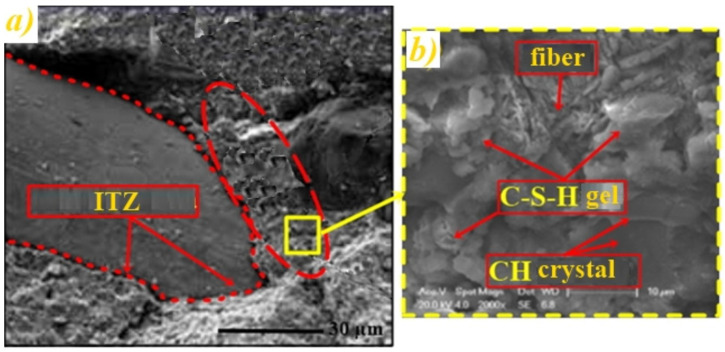
Interfacial transition zone [111]: (**a**) Wide SEM micrograph of the sample (**b**) Amplification of the SEM with effect of fiber bonding and the compaction of the interfacial transition zone (ITZ).

One of the most effective techniques for investigating the distribution, adhesion and compatibility between the reinforcement and the composite matrix is scanning electron microscopy (SEM). It is one of the most commonly used ways to analyze the interfacial interaction between reinforcement and matrix, which leads to a positive effect on both tensile and shear strengths [104]. As an example, Figure 3 shows the SEM micrograph of a fiber-reinforced composite showing the effect of fiber bonding and the compaction of the interfacial transition zone (ITZ), as indicated by Fediuk et al. [111].

## 4. Durability, Chemical Degradation and Other Properties

The main disadvantage of NLFs when used in composites is their durability in the matrix, especially in cementitious matrices, due to their application in the vast majority of Portland cement composites. In this type of binder, hardening occurs through hydration reactions of anhydrous compounds, originating hydrated calcium silicate, ettringite and calcium hydroxide. Calcium hydroxide forms during hydration, together with other alkalis that may be present in the matrix such as potassium and magnesium hydroxide, migrates to the fiber walls, causing mineralization, resulting in a loss of toughness and strength and compromising the durability of the composite [46,47,112].

There are two mechanisms of mineralization: that by calcium hydroxide and self-to-mineralization. The degradation process of natural fibers occurs in the cement matrix in four alternating steps, namely: (i) degradation of lignin and part of hemi-cellulose leading to exposure of holocellulose; (ii) degradation of hemicellulose causing decreased integrity and stability of cell walls; (iii) degradation of intramolecular hydrogen bonding leading to dispersion of cellulose microfibrils; and finally, (iv) alkaline hydrolysis of amorphous regions and complete degradation of cellulose microfibrils [48]. 

According to Bentur and Akers [113], some mechanisms can change the behavior of composites reinforced with natural fibers regarding durability and mechanical properties. These are: (i) alkaline hydrolysis of cellulose molecules, leading to a reduction in the degree of polymerization and lower tensile strength; (ii) dissolution of lignin and hemicelluloses, breaking the bonds between the fiber cells; (iii) an increase in fiber-matrix adhesion that can lead to embrittlement; and (iv) microbiological attack, whose occurrence is more likely in less alkaline matrices.

NFLs in an alkaline environment, such as those in the cementitious matrix, undergo an aging process that results in the loss of tenacity and strength in post-cracking. This durability problem is associated with increased fiber breakage and pullout [114]. This is generally due to three factors: alkali attack, fiber mineralization and volume variation. The alkali attack causes the chemical decomposition of lignin and hemicellulose present in the middle lamella, breaking the bond between the microfibrils. Mineralization is the migration of hydration products, mainly calcium hydroxide, to the surface and lumen of the fiber. The crystallization that occurs decreases the fiber’s strength and flexibility. The volume variation occurs due to the high water absorption of the fiber, resulting in the weakening of the fiber-matrix interface [114,115].

Some strategies can be used to alleviate the problem of durability of NLFs. These include modifying the fibers through impregnation with repellents and water blocking agents, and with microsilica paste [116,117]; and modifying the matrix by sealing the pores [115] or reducing its alkalinity [114,116,118]. 

To reduce alkalinity, part of the Portland cement is normally replaced by pozzolanic materials, such as blast furnace slag, microsilica, calcined clay or metakaolin. Pozzolanic materials have a high content of amorphous siliceous compounds that consume calcium hydroxide, forming hydrated calcium silicate in an alkaline environment. This reaction keeps the matrix low in calcium hydroxide, and efficiently combats the aging process of natural fibers [114,116,119,120].

Another aspect of the matrix that is influenced by the insertion of NLFs is rheology. There is a loss of consistency and workability due to the increase in the water absorption surface area, owing to the hydrophilic character and intrinsic porosity of the fibers. The higher the fiber aspect ratio and its volumetric fraction, the worse the workability of the pulp [121]. Despite this, it is essential to maintain workability, to ensure adequate compaction when used with discrete fibers and to penetrate between the strands of fabrics. One option is to treat the fibers earlier to reduce water absorption or to wet them before adding to the matrix. Furthermore, water absorption can be taken into account during the matrix trace dosing process [122].

The use of NLFs in cementitious matrices, in addition to decreasing the workability of the paste, delays the setting or hardening time [122]. According to Sedan et al., the explanation for this behavior is that pectin can adsorb calcium, preventing the formation of hydrated calcium silicate. The American Concrete Institute (ACI) suggests using its Type III Portland cement (high early strength cement) to contribute to the setting process [122].

## 5. Properties of the Composite with Amazon NLFs

The purpose of using NLFs in cementitious composites is to improve the properties of an inherently weak, brittle, and crack-prone cementitious matrix [100]. These improvements depend on the type and content of fiber used in the composite, with a view to improving the properties in the fresh state and in the hardened state of cementitious composites. An approach to such properties in both states is provided in this section. For this, a compilation of recent studies from different authors on diverse Amazon NLFs is presented in Table 4.

According to Marvila et al. [100], the incorporation of any type of NLF in concrete reduces its workability, with the loss being proportional to the fiber volume concentration in the composite. Azevedo et al. [52] studied Portland cement mortars reinforced with açai fibers in a 1:1:6 ratio. Proportions of 1.5%, 3% and 5% fiber were added in relation to the cement mass. These variations were used for untreated fibers, as well as for those treated by immersion in alkaline solution at 5% concentration in NaOH. The authors observed in their research that the addition of an NLF, as well as increasing the percentages, reduces the workability of the mortar. This reduction is due to the greater mass stability that the addition of fiber gives, providing greater internal cohesion of the constituents and filling gaps. The surface treatment created fibers with great roughness, generating an interfacial adhesion between the reinforcement (fiber) and the matrix (mortar), which corroborates for an even greater reduction in the fluidity of the mortar due to the anchorage of the fiber in the matrix, thus affecting the workability.

Azevedo et al. [124] investigated cementitious composites reinforced with curaua fibers in a 1:1:6 ratio. Specimens were produced with 1%, 2% and 3% in the untreated condition and in the condition treated under mercerization with NaOH. The reduction in consistency observed in Table 4 indicates the difficulty for the mortars to spread; that is, they indicate a reduction in the workability of the material, attributed to the absorption of water by the untreated fibers, which retain water in their composition, impairing the presence of water free in the mortar air and the fluidity of the material. In the case of treated fibers, as there was a reduction in the water absorption of the material, a smaller reduction in the consistency of the mortars was observed; that is, the treatment of curaua fibers improves the workability of the mortar when compared to the non-adherent fibers treated.

The water retention of the mortar is reflected in the coating properties. A mortar with low water retention has high exudation, impairing the workability of the mortar and the mechanical resistance of the coating, especially the surface resistance due to lack of water for the hydration of the cement, owing to excessive loss to the environment. In concrete or mortar structures, if the amount of water lost per unit of area exposed to the environment is greater than the amount that rises to the surface due to the exudation effect, the appearance of cracks due to plastic shrinkage may occur [128].

Toledo Filho and Sanjuán [129] demonstrated in their studies that sisal fibers are extremely effective at decreasing the plastic shrinkage of mortars by reducing the time for the appearance of the first crack and in controlling cracking, when compared to mortars with polypropylene fibers. According to the authors, this is due to the fact that, in the early stages, the modulus of elasticity of these fibers is still greater than that of the cementitious matrix.

Due to their hydrophilic character, natural fibers have a great water retention capacity, affecting the water available in the hydration of the cement paste (matrix), which impairs several properties of the compound. Azevedo et al. [52] verified that the addition of natural açai fibers in a cementitious matrix without any type of treatment reduces the water retention because the water is internally stored in NLFs. As an alternative, surface treatment with NaOH reduced water retention in açai fibers compared to the reference mix (without any fiber).

The incorporated air content is related to the mass density of the mortar in its fresh and hardened state. As the air content increases, the mortars become less dense. The more air incorporated, the more effects are seen in the ability of the mortar to deform and the greater influence on the launch energy and mechanical properties. Air bubbles in the cementitious matrix can be introduced by air-incorporating additives, or in the case of fiber-reinforced mortars, by trapping voids resulting from the mixing process [128].

Azevedo et al. [52] also found that the addition of untreated natural fiber increases the in-built air content. This is justified by the increase in the contact surface area (interface zone) between the matrix and the reinforcement as the percentage of fiber addition increases, favoring the formation of voids, which are filled with air or water, depending on the moisture content of the paste.

The mechanical characteristics of the mortar, although they do not faithfully reflect the characteristics of the coating, must be known so that it can be inferred how the mortar may behave when applied as a coating. In addition, these characteristics can verify whether the coating’s internal consolidation state is capable of withstanding mechanical actions of the most diverse natures. Indeed, they seek to reproduce, in the mortar specimen, the type of mechanical action that occurs inside the coating, as is the case for the traction and compression effort, in view of, for example, temperature variations that occur in the environment. Thus, from the determination of the mortar’s tensile strength, it is possible to expect that the mortar will be able to withstand the efforts acting during the movement of the coating. It also allows deducing other properties that are directly related to mechanical strength, such as the compressive stress. Moreover, its magnitude can give an idea of the static deformation modulus, and infer the tightness and weatherability of the coating [12].

In the study by Azevedo et al. [124] with cementitious composites reinforced with curaua fibers, it was observed that the use of these fibers without treatment considerably reduces the tensile strength of the composites. This can be attributed to the presence of impurities such as waxes, ash and sugar that delay or inhibit hydration reactions. They can also be related to the presence of entrained air, as already discussed. When using treated fibers, however, there is an effective increase in the tensile strength of the composites, attributed to the transfer of deformation from the matrix to the fibers. Regarding the compressive strength, the result obtained can be explained by the fact that the matrix cement mortar breaks with the formation of cracks parallel to the compressive stresses. Each fiber prevents the development of micro-cracks and thus increases the compressive strength.

Although the flexural strength after the incorporation of natural fibers shows increased nominal values in the literature, Azevedo et al. [52] observed, in the case of natural açai fiber, that the values decrease because these fibers, despite reinforcing the composite, are very short. The shorter length causes slippage due to lower interfacial adhesion tension between reinforcement and padding. After the surface treatment of the açai fibers with NaOH solution, the tension is increased, improving the adhesion mechanisms that corroborate with the greater resistance to bending. In the case of the 5% addition, the excess of fibers immersed in the filling, even when treated, create rupture in preferential areas, reducing flexural strength. On the other hand, the addition of açai fibers provides a characteristic increase in compressive strength due to greater compaction of the internal matrix, proven by the reduction in capillary absorption.

When evaluating the results found in the study by Thomas and Jose [127] on cementitious composites with sisal fiber, it is clear that the mechanical property of sisal fibers is entirely reliant on the manufacturing situation, as well as the sisal fiber size. The dimension was the one that attempts to determine the character property, fiber parameters, length, environmental aspect, strain rate, and so on. On the other hand, the sisal fiber displays high ductility within the fracturing region of the specimens. Increasing use of NLFs in the concrete mixture promotes significant control of the cracks and the breakage. 

Finally, the bamboo fiber studied by Correia et al. [123] showed mechanical results of composites reinforced with 6% and 8% of the fiber with higher tension than those reinforced with 10% and 12% of the fiber. The mechanical performance of the matrix of composites reinforced with 10% and 12% of the fiber was lower due to the excess porosity added by the fiber, while the tenacity in the post-cracking conditions was higher. The ideal level of reinforcement must be selected based on the application of the composite; that is, if the application of the material requires greater mechanical strength or greater impact resistance.

## 6. Practical Applications of Cementitious Composites Reinforced with NLFs

An important aspect to be addressed in a review such as this is the practical applicability of cementitious composites reinforced with NLFs. Currently, cementitious composites, which can be used as mortar or concrete, have a wide range of applications, and some of them will be dealt with in this section.

For long-term soil stabilization, for example, a cementitious composite reinforced with chemically treated NLFs is considered a good solution. Sarsby [130] was of the opinion that for the transitional separation of subsoil and subbase in road construction, erosion control and backfill support, natural fibers such as coconut, sisal and flax may outperform those of synthetic geotextiles. In a related study, a substantial improvement in the ductility of a flax fiber-reinforced soil-cement composite was reported by Segetin et al. [131]. They further suggested that spray pre-coating these fibers could improve mechanical properties.

Cement composites incorporated with NLFs are also used as an internal curing agent and can therefore be applied onto the surface for the curing of concrete infrastructures. Some studies [132,133] have shown that lane closures associated with traditional methods of curing shotcrete repairs on bridge soffits could be avoided through the use of wet-sprayed cellulose pulp fibers.

Concerning applications in the form of concrete, [134] investigated a jute textile for concrete reinforcement, finding a high potential of this new material for the development of thin walled elements. The applications of these composites in mortar to reinforce structures are also widespread. Teixeira e Silva [135] developed a mortar reinforced with curaua fiber that has excellent behavior as a structural component, providing greater load and deformation capacity to the reinforced beam.

## 7. Final Remarks

Relating the properties presented by cementitious composites reinforced with Amazon NLFs (Table 4) and their practical applications is the objective of this section.

The workability of cementitious material is the property in the fresh state that determines the ease and homogeneity with which it can be mixed, cast, compacted and finished. It is clear that workability is one of the main properties, since it interferes with the way that the material is deposited in the fresh state. All practical applications of a cementitious material depend on a certain degree of workability to be viable for use, especially when it comes to mortars and concrete. Molding and densifying a concrete with little workability becomes an action that is difficult to perform and can generate execution failures, leading to pathologies. Evaluating the results presented here, it is noticed that with the increase of the fiber content in the mixture, a lower workability is achieved. Therefore, the incorporation of any type of NLF in the cementitious material reduces the workability, the loss being proportional to the concentration of the fiber volume in the composite [100]. This fact is due to the greater mass stability that the addition of fiber confers, providing greater internal cohesion of the constituents and filling gaps.

In addition, the workability is also impaired by the absorption of water by untreated NLFs, which retain water in their composition, impairing the presence of free water in the air of the mortar and the fluidity of the material. In the case of the treated fibers, as there was a reduction in the water absorption of the material, there was a smaller reduction in the consistency of the mortars. Therefore, the treatment of the fibers is a mechanism to attenuate the loss of workability of the mortar when compared to the untreated fibers.

It is known that cement is a hydraulic binder, so it needs water to form hydration reactions with the clinker constituents and obtain mechanical strength. Therefore, it is necessary that the mixing water added to the mixture remains in the composite and is not lost to the environment, nor to other materials such as blocks and bricks. This justifies the water retention property, where mortars and concretes with high water retention are ideal for use as they will have enough water retained for the hydration reactions. The mortar’s water retention property is reflected in the coating properties, where a mortar with low water retention presents high exudation, impairing the mortar’s workability and the mechanical resistance of the coating, mainly the surface resistance due to the lack of water to cement hydration, caused by excessive loss to the environment. In concrete or mortar structures, the phenomenon of exudation can generate the appearance of cracks by plastic shrinkage [128]. Practices such as structural repair works, infrastructure and bridges can be highly affected by the shrinkage phenomenon. NLFs have a high water retention capacity, affecting the water available in the hydration of the cement paste, which impairs several properties of the compound. Analyzing the data presented in Table 4, it can be seen that the addition of NLFs to a cement matrix without any type of treatment reduces water retention, as water is retained internally in the fibers. Alternatively, surface treatment reduces water retention in the fibers.

Concerning the mechanical strength of fiber-reinforced cementitious composites, Souza et al. [75] report that fibers in cementitious materials can have at least three effects on the hardened state of these composites. The first effect is the tendency to increase the stress under which the matrix cracks, which is more evident under loads that generate direct tension, bending and shear, directly affecting practices such as soil stabilization and cladding mortars. The second effect is the improvement of the deformation capacity of brittle materials, due to an increase in the energy absorption capacity or toughness. The third effect is the ability to inhibit or modify the development of cracks by reducing the opening and spacing between them. This effect depends on the type and amount of fibers incorporated, in addition to the cracking mechanism that occurs. The last effect predicts important improvements for practical applications in infrastructure, bridges, reinforced concrete in general, but mainly in thin-walled structures, where the shrinkage phenomenon can be more intense. Evaluating the data presented in Table 4, it can be seen that the use of untreated NLFs considerably reduces the tensile strength of composites. This can be attributed to the presence of impurities such as waxes, ash and sugars that delay or inhibit hydration reactions. On the other hand, with the use of treated fibers, there is an effective increase in the tensile strength of the composites, due to the transfer of deformation from the matrix to the fibers. As regards the compressive strength, the matrix breaks with the formation of cracks parallel to the compressive stresses, where each fiber prevents the development of microcracks and thus increases the compressive strength.

## 8. Conclusions

The main objective of this work was to compile and discuss important research that has used Amazon NLFs as reinforcement for cementitious materials, approaching the advantages and disadvantages of its application as well as challenges in the processing and exploitation of these natural resources.

Regarding the feasibility of the application of Amazon NLFs in cementitious materials, the great potentiality of these fibers in this type of composite can be noticed, since innumerable practical techniques are available to improve the material used for these purposes. Applications such as soil stabilization, infrastructure, bridges, reinforced concrete, structural reinforcement mortars and even the production of thin concrete walls have been reported [130,131,132,133,134,135]. The studies prove that the gain in the relevant properties for their due purposes when using Amazon NLFs in cementitious materials makes the material promising.

In addition, in those studies it was possible to understand the particularities related to NLFs from the Amazon region. The main properties of these fibers are highlighted as physical, mechanical and chemical properties.

As for the physical properties, density stands out Table 2, where the vegetable fibers present values lower than those of the matrix, reducing the final weight of the composite and the high water absorption of the vegetable fibers, reaching values above 100%.

In terms of the mechanical properties, the tensile strength stands out, with NLFs showing high values, thus improving the tensile property in cementitious materials. Furthermore, the incorporation of fibers in the composite can have three effects: (i) a tendency to increase the tension under which the matrix cracks, which is more evident under loads that generate direct traction, bending and shear efforts; (ii) improvement in the deformation capacity of fragile materials, due to the increase in the energy absorption capacity or toughness; and (iii) the ability to inhibit or modify the development of cracks by reducing the openings and spacing between them.

Regarding chemical properties, it is observed that vegetable fibers are characterized by being predominantly composed of cellulose, lignin, hemicellulose and pectin, with smaller amounts of wax, ash and sugars, which must be removed from the fibers to allow for their application in cementitious matrices. It is noteworthy that cellulose is the compound responsible for the fiber’s strength, while lignin and hemicellulose are responsible for its low durability. Therefore, it is necessary to carry out treatments, usually with alkaline solutions, to improve the adhesion of the matrix with the fiber in the interface region. It is possible to verify the difference in behavior of the treated and untreated reinforcement material in the results of the composite properties. In general, treated fibers, which have a better adhesion mechanism in the interface zone, reveal lower workability losses, less water retention inside the hydrophilic fiber, and higher mechanical strength than cement composites reinforced with untreated Amazon NLFs.

Furthermore, NLFs treatments provide relief from the durability problem caused by the use of fibers in an alkaline medium, such as the cement matrix, reducing problems such as loss of tenacity and post-cracking strength. Thus, fiber treatment and addition of pozzolan to the composite are effective ways to improve the durability of the cement composite reinforced with natural fibers and, finally, generate applicability for this material.

In this work, the use of Amazon NLFs, such as açai, jute, sisal, bamboo, and curaua, in cementitious matrices was emphasized. However, many Amazon NLFs already characterized and studied in other matrices have not been completely evaluated in cementitious materials. Thus, further investigations with guaruman and piassava fibers in cementitious composites are suggested as future works.

Thus, these topics are suggested for future works:

- Application of guaruman and piassava fibers in cementitious matrices;
- Application of other alkaline treatments, such as potassium and magnesium hydroxide, on vegetable fibers;
- Standardization of vegetable fiber treatments for application in cementitious matrices;
- Studies of new applicability in composite building materials reinforced with Amazon NLFs.

## Figures and Tables

**Table 1:** Chemical properties of Amazon NLFs.

Fiber Type	SCIENTIFIC NAME	Cellulose (%)	Lignin (%)	Hemicellulose (%)	Pectin (%)	Wax (%)	Ash (%)	Moisture Content (%)	Reference
Açai	*Euterpe oleracea*	45–47	31–34	17–20	-	-	-	10	[52]
Bamboo	*Bambusa vulgaris*	33–45	20–25	21–30	13–14	1	4–5	12	[57,58]
Curaua	*Ananas erectifolius*	71–74	9–11	21–26	-	1–2	2–3	18	[53,54]
Guaruman	*Ischnosiphon koem*	39–40	10–12	40–41	-	-	7–9	13	[55]
Jute	*Corchorus capsularis*	45–71	12–26	14–21	1–10	1	1–2	12	[53,59]
Piassava	*Attalea funifera*	29–32	45–48	26	-	-	-	14	[56]
Sisal	*Agave sisalana*	65–75	8–12	10–15	2–10	1–2	1–2	11	[53,59]

**Table 3:** Mechanical properties of Amazonian fibers.

Fiber Type	Tensile Strength (MPa)	Modulus of Elasticity (GPa)	Reference
Açai	17.8	15.7	[52]
Bamboo	140–800	27	[56,91]
Curaua	488–752	31.8–64	[56,75]
Guaruman	614	21	[78]
Jute	393–773	26.5	[80,92]
Piassava	43–79	1.36–2.28	[76]
Sisal	511–635	9–22	[80,93]

**Table 4:** Properties of the composite with Amazonian fibers.

Fibers Type	Addition Percentage	Consistency (mm)	Content of Incorporated Air (%)	Water Retention (%)	Tensile Strength (MPa)	Compression Strength (MPa)	Reference
Açai	1.5% Untreated Fiber	255	7.5	95.16	1.13	3.72	[52]
	3% Untreated Fiber	228.33	7.9	94.41	1.03	4.02	
	5% Untreated Fiber	218	8.4	93.37	0.97	3.83	
	1.5% Treated Fiber	211	7.3	95.61	1.54	3.84	
	3% Treated Fiber	222.45	7.7	95.11	1.76	4.23	
	5% Treated Fiber	207	8.2	95.07	1.42	3.94	
Bamboo	6%	-	-	-	6.4 ± 0.9	-	[123]
	8%	-	-	-	7.5 ± 0.1	-	
	10%	-	-	-	6.8 ± 1.4	-	
	12%	-	-	-	5.8 ± 1.5	-	
Curaua	1% Untreated fiber	257.23 ± 2.33	8.23 ± 0.31	95.46 ± 1.08	3.1 ± 0.1	4.4 ± 0.15	[124]
	2% Untreated fiber	253.21 ± 1.67	8.25 ± 0.20	97.42 ± 0.65	3.0 ± 0.2	4.25 ± 0.1	
	3% Untreated fiber	249.44 ± 1.80	8.98 ± 0.18	98.89 ± 0.81	2.9 ± 0.2	4.2 ± 0.15	
	1% Treated fiber	261.22 ± 0.97	7.92 ± 0.27	92.34 ± 0.33	3.7 ± 0.2	6.8 ± 0.4	
	2% Treated fiber	257.54 ± 2.01	7.84 ± 0.35	94.45 ± 0.67	3.9 ± 0.17	7.0 ± 0.2	
	3% Treated fiber	254.23 ± 2.12	7.80 ± 0.24	95.67 ± 0.56	3.9 ± 0.2	7.0 ± 0.15	
Guaruman	2.5% Untreated fiber	249.87	7.9	94.56	-	-	[55]
	5% Untreated fiber	252.43	8.5	93.4	-	-	
	7.5% Untreated fiber	209.87	8.8	92.7	-	-	
	2.5% Treated fiber	262.48	7.7	95.2	-	-	
	5% Treated fiber	246.74	8.15	94.98	-	-	
	7.5% Treated fiber	215.63	8.3	94.24	-	-	
Jute	1.5%	-	-	-	5.71	56.45	[125]
Piassava	2% Untreated fiber	257.6	8.1	94.96	-	-	[126]
	5% Untreated fiber	246.4	8.7	93.8	-	-	
	2% Treated fiber	260.2	7.9	95.37	-	-	
	5% Treated fiber	248.9	8.3	94.46	-	-	
Sisal	1% Treated Fiber	-	-	-	2.25	21	[127]
	1% Treated Fiber	-	-	-	2.75	18	
	1.5% Treated Fiber	-	-	-	2.6	16	
	2% Treated Fiber	-	-	-	2.4	19	
	2.6% Treated Fiber	-	-	-	1.8	19.5	

## Data Availability

Not applicable.
